# Identification of lymph node metastasis-related genes and patterns of immune infiltration in colon adenocarcinoma

**DOI:** 10.3389/fonc.2022.907464

**Published:** 2023-01-16

**Authors:** Haoxiang Zhang, Guibin Zhao, Guangwei Zhu, Jianxin Ye

**Affiliations:** ^1^ Department of Gastrointestinal Surgery 2 Section, Institute of Abdominal Surgery, Key Laboratory of Accurate Diagnosis and Treatment of Cancer, The First Affiliated Hospital, Fujian Medical University, Fuzhou, China; ^2^ Department of Gastrointestinal Surgery 2 Section, National Regional Medical Center, Fujian Medical University, Fuzhou, China; ^3^ Department of Gastrointestinal Surgery, Mindong Hospital Affiliated to Fujian Medical University, Fuan, China; ^4^ Key Laboratory of Ministry of Education for Gastrointestinal Cancer, Fujian Medical University, Fuan, China

**Keywords:** immune cell infiltration, lymph node metastasis, TCGA, bioinformatic analysis, colon adenocarcinoma

## Abstract

**Backgrounds:**

Colon adenocarcinoma(COAD) is one of the most common tumors of the digestive tract. Lymph node metastasis (LNM) is a well-established prognostic factor for COAD. The mechanism of COAD lymph node metastasis in immunology remains unknown. The identification of LNM-related biomarkers of COAD could help in its treatment. Thus, the current study was aimed to identify key genes and construct a prognostic signature.

**Methods:**

Gene expression and clinical data were obtained from The Cancer Genome Atlas (TCGA) database. Differentially expressed genes were calculated by using R software. GO functional and KEGG pathway enrichment analysis were processed. The CIBERSORT algorithm was used to assess immune cell infiltration. STRING database was used to screen key genes and constructed a protein-protein interaction network (PPI network). The LASSO-Cox regression analysis was performed based on the components of the PPI network. The correlation analysis between LNM-related signature and immune infiltrating cells was then investigated. TISIDB was used to explore the correlation between the abundance of immunomodulators and the expression of the inquired gene.

**Results:**

In total, 394 differentially expressed genes were identified. After constructing and analyzing the PPI network, 180 genes were entered into the LASSO-Cox regression model, constructing a gene signature. Five genes(PMCH, LRP2, NAT1, NKAIN4, and CD1B) were identified as LNM-related genes of clinical value. Correlation analysis revealed that LRP2 and T follicular helper cells (R=0.34, P=0.0019) and NKAIN4 and T follicular helper cells (R=0.23, P=0.041) had significant correlations. Immunologic analysis revealed that LRP2 and NKAIN4 are potential coregulators of immune checkpoints in COAD.

**Conclusion:**

In general, this study revealed the key genes related to lymph node metastasis and prognostic signature. Several potential mechanisms and therapeutic and prognostic targets of lymph node metastasis were also demonstrated in COAD.

## Introduction

Colorectal cancer (CRC) is the third most commonly diagnosed neoplasm, with the second-highest cancer-associated mortality rate globally (1). Colorectal cancer includes cancers of both the colon and the rectum and is the most common form of gastrointestinal cancer. About 90% of colorectal cancers are adenocarcinomas originating from the colorectal mucosal epithelium in the world (2). The treatment of colorectal cancer includes surgery, chemotherapy, radiotherapy, and other biological, immunological treatments. Although therapies for colorectal cancer have improved in recent years, the survival rate of colorectal cancer patients is still not satisfactory (3). The development of a novel therapeutic target is required to increase the survival rates of colorectal cancer patients. Therefore, we conducted a genetic and immunologic study on colon adenocarcinoma(COAD).

Lymph node metastasis (LNM) is an independent risk factor affecting the prognosis of patients with COAD (4–6). The extent of lymph node metastasis affects the postoperative staging and subsequent treatment measures of COAD patients. The TNM system of the American Joint Committee on Cancer/International Union Against Cancer Classification (AJCC/UICC) recommends a minimal assessment of 12 lymph nodes for accurate staging. Currently, some studies have investigated the mechanism of COAD lymph node metastasis. The combination of p53 deficiency and azoxymethane (AOM) promotes tumor development, including the growth of invasive cancers and lymph node metastasis (7). Smad4 and VEGF-C are involved in lymphangiogenesis and lymphatic metastasis (8). FHL2 overexpression of myofibroblasts in sporadic colon cancer was significantly linked to lymphatic metastasis (9). The tumor microenvironment of COAD is associated with tumor lymph node metastasis. Interferon regulatory factor 1 (IRF1) was associated with tumor stage, lymph node metastasis, and distant metastasis. Furthermore, there are significant associations between IRF1 and CD8+ T cells, T cell, dendritic cells, T-helper 1 cells, and T cell exhaustion (10). It appears that the mechanism of lymph node metastasis in COAD is complicated and is related to immune-related genes and tumor microenvironment.

We conducted a study on lymph node metastasis-related genes(LNM-related genes) in COAD and revealed the relationship between LNM-related genes and immune infiltrating cells in COAD. In our study, datasets for colon adenocarcinoma samples were downloaded from The Cancer Genome Atlas (TCGA) public database. We screened out the differentially expressed genes between N+ (COAD patients with lymph node metastasis) and N0 (COAD patients without lymph node metastasis) samples and constructed a protein-protein interaction network(PPI) to screen out LNM-related genes to construct a survival prognosis model. The co-expression analysis of genes with prognostic significance and immune infiltrating cells of COAD was conducted to illustrate the potential mechanisms of lymph node metastasis in COAD.

## Materials and methods

### Identification of differentially expressed genes

**Figure 1:**
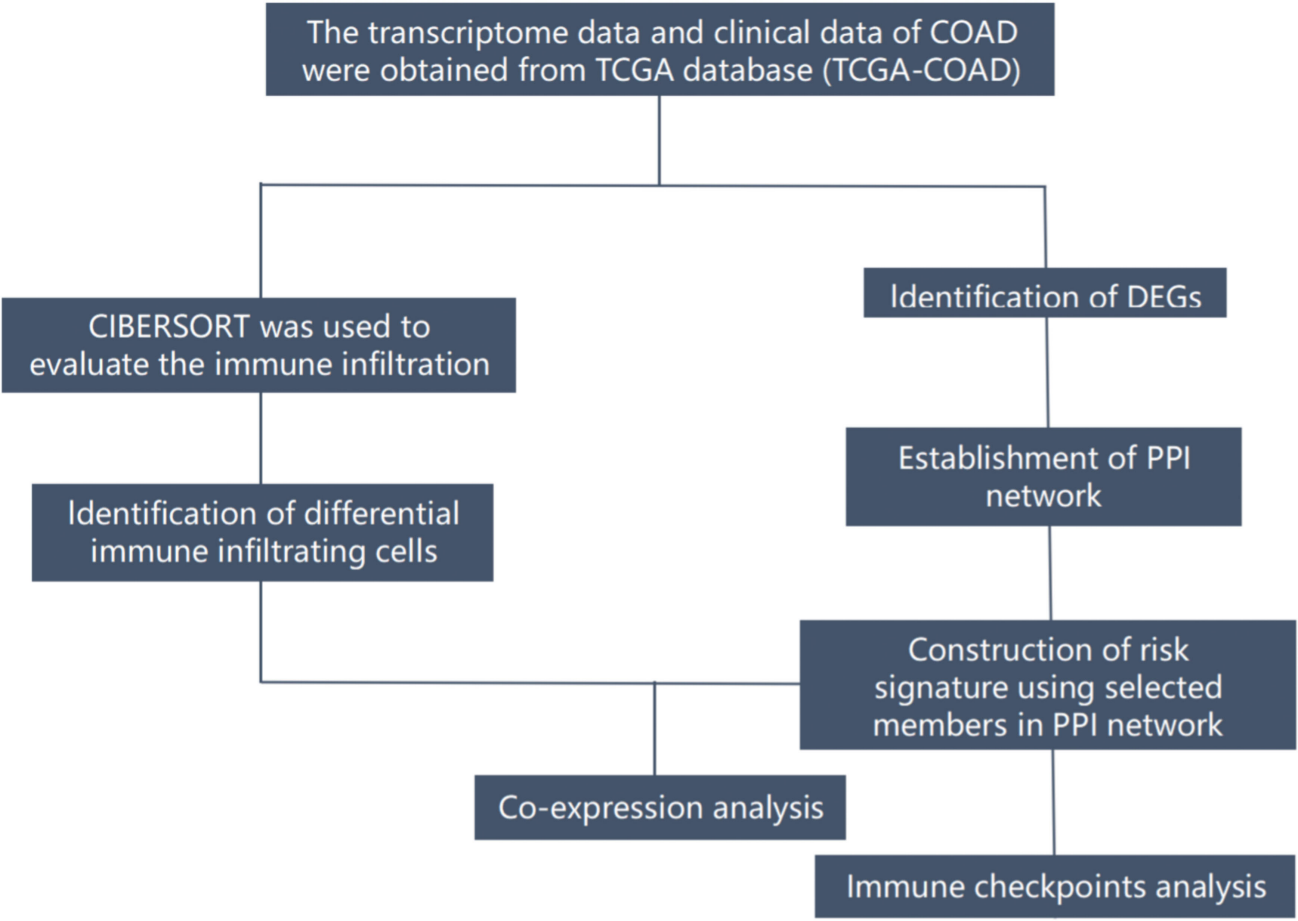
The flow diagram of the present study. COAD, colon adenocarcinoma; DEGs, differentially expressed genes; TCGA, The Cancer Genome Atlas; PPI, ptotein-protein interaction;.

COAD (TCGA-COAD) RNA-seq data were downloaded from the TCGA public data platform(The Cancer Genome Atlas) (https://portal.gdc.cancer.gov/), including 473 cases of COAD tumor tissues and 41 cases of normal tissues. Normal tissue data were eliminated, and selected high-quality samples in duplicate samples(1. Frozen tissue is preferred to formalin-fixed paraffin-embedded tissue; 2. Tissue with larger plate value). Finally, 447 tumor tissue with clinical information were selected for the identification of differentially expressed genes. Demographic and clinical data of 447 patients with COAD are summarized(including age, gender, stage, and TNM classification) (**Table 1**). Tissue samples were divided into N+ group (n=181) with positive lymph node metastasis and N0 group without lymph node metastasis (n=266). Limma R package (version: 3.40.2) of R software was used for differential expression analysis between the two groups. The adjusted P-value was analyzed to correct for false-positive results in TCGA. FDR< 0.05 and |log2 fold change|> 1 were defined as the thresholds for differential expression analysis. A graphical representation of the complete workflow is provided in **Figure 1**.

**Table 1:** Characteristics of the included COAD patients obtained from the TCGA database.

Basic information	N+ group (n=181)	N0 group(n=266)
Age	66 (median)	70 (median)
Gender		
Female	91	145
Male	90	121
AJCC Stage		
I & II	0	251
III & IV	177	8
Unknow	4	7
T classification		
Tis	0	1
T1 & T2	9	77
T3 & T4	172	188
N classification		
N0	0	266
N1	102	0
N2	79	0
M classification		
M0	102	228
M1	53	8
MX	26	30

COAD; colon adenocarcinoma; TCGA, the The Cancer Genome Atlas; AJCC, American Joint Committee on Cancer.

To further confirm the underlying function of potential targets, the data were analyzed by functional enrichment. Gene Ontology (GO) is a widely-used tool for annotating genes with functions, especially molecular function (MF), biological pathways (BP), and cellular components (CC). Kyoto Encyclopedia of Genes and Genomes (KEGG) Enrichment Analysis is a practical resource for analyzing gene functions and associated high-level genome functional information. To better understand the carcinogenesis of mRNA, the ClusterProfiler package (version: 3.18.0) in R was employed to analyze the GO function of potential targets and enrich the KEGG pathway. Ethical approval from an institutional review board was not required because the survey data are publicly available. The study was conducted following the Declaration of Helsinki (as revised in 2013).

### Construction of a protein-protein interaction network

STRING is a public database that contains interactions between known and predicted proteins (https://string-db.org/). The database covers protein interactions from 2031 species containing a total of 9,643,763 proteins and 1,380,838,440 interactions. In addition to the experimental data, the results of text mining from PubMed abstracts and the synthesis of other database data are also included in the database. It also uses bioinformatics methods to predict the results. The PPI network was constructed by the STRING database based on differentially expressed genes between the N+ group and the N0 group. The PPI network was then visualized using Cytoscape software version 3.8.0 (www.cytoscape.org/).

### Survival analysis based on the components of the PPI network

All components of the PPI network were incorporated into the Cox regression model, and to assure multifaceted models were not overfitting, the Lasso regression was used. All patients were consequently classified into a high- and low-risk group by utilizing the median risk score calculated by LASSO as the cutoff value. Kaplan-Meier survival curves were compared using the log-rank test. Receiver operating characteristic (ROC) curves were constructed to estimate the area under the ROC curve. A genomic nomogram was developed based on the LASSO-Cox regression model analysis results to predict the 1, 3, 5-year overall survival(OS). The nomogram provided a graphical representation of the factors, which can calculate the risk of death for an individual patient by the points associated with each risk factor through the “rms” R package. The calibration curve was used to evaluate the consistency/calibration, that is, the difference between the predicted and true values. Genes with prognostic significance in the PPI network are defined as lymph node metastasis-related genes (LNM-related genes) in COAD.

### Immune cell infiltration

We analyzed immune cell infiltration in each tumor sample using the CIBERSORT method (p < 0.05). CIBERSORT is a deconvolution algorithm that can predict the abundance of 22 immune cell types based on the gene expression data. WilcoxTest was used to screen the differential immune cell infiltration between the N0 and N+ groups.). Then, Pearson’s correlation coefficient was used to assess the correlations between the differential immune infiltrating cells and LNM-related genes.

### Immunologic analysis

The investigated immunoinhibitors were collected according to Garraway et al.’s study (11–15). Based on five studies, we selected immune checkpoints with the top ten mutation rates. Pearson’s correlation analysis was used to evaluate the relationship between LNM-related genes and immune checkpoints. Each Spearman correlation between inquired gene and a distinct immunoinhibitor in an individual cancer type was integrated into the indicated heatmap.

### Statistical analysis

All statistical analysis were performed using R 4.11 software. PPI network was constructed by “STRING.” Univariate and multivariate analysis were performed with log-rank test and Cox regression, using the survival package of R. There were two outcome variables (status and survival time). The expression of the components of the PPI network and immune cell infiltration were assumed to be independent variables in Cox regression used for two survival analysis. LASSO-Cox regression model analysis was performed using R software’s package “glmnet” (version 4.11). Receiver operating characteristic (ROC) curves of 1, 3, 5 years were plotted, and the area under the curve (AUC) values were calculated using the R package “survivalROC.” Nomograms and calibration plots were done with “rms” package of R software. Differences in gene expression between the two groups were evaluated using the Mann-Whitney U-test.

## Results

### DEGs

**Figure 2:**
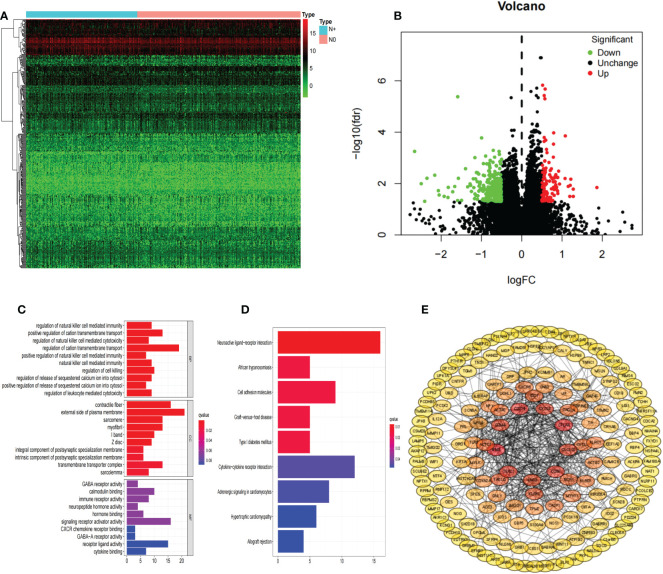
DEGs. Hierarchical clustering analysis of genes **(A)**; Volcano plots were constructed using fold-change values and adjusted P **(B)**; Red dots represent upregulated genes, and green dots represent downregulated genes. The GO analyses **(C)**; KEGG pathway analyses **(D)**; PPI network of differentially expressed genes **(E)**. DEGs, differentially expressed genes; COAD, colon adenocarcinoma.

We identified 394 differentially expressed genes classified as upregulated (115) or downregulated (279) in the COAD N+ group versus N0 group tissues (**Figures 2A, B**). We next analyzed DEGs by GO and enrichment in KEGG pathways (**Figures 2C, D**).

### PPI network construction and survival analysis

**Figure 3:**
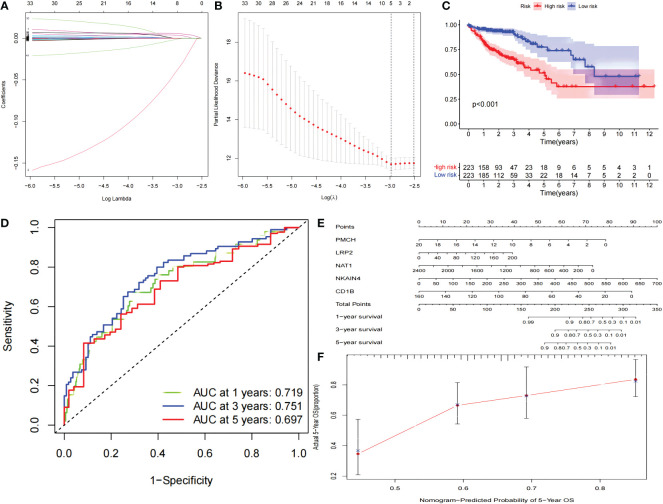
The PPI network signature. The PPI network signature was constructed based on the components of the PPI network associated with survival according to LASSO-Cox regression analysis **(A, B)**. Kaplan-Meier survival curve **(C)**, The results of ROC curve **(D)**, nomogram **(E)**, and calibration curve **(F)** analyses are shown. LASSO, least absolute shrinkage and selection operator; ROC, receiver operating characteristic; PPI, protein-protein interaction.

A PPI network was constructed composed of 180 DEGs based on the STRING database (**Figure 2E**). Univariate Cox proportional hazards regression analysis, LASSO logistic regression analysis, multivariate Cox regression analysis model were used to screen out the characteristic genes associated with survival (**Figures 3A, B**). The P-value, HR, and 95% CI of each variable are shown in **Table 2**. The performance of the risk score was evaluated by dividing the COAD samples in the TCGA into two subgroups, high-risk and low-risk, using the median risk score (1.14) calculated by LASSO as a cutoff. Risk stratification by risk score showed that patients in the high-risk subgroup had a shorter OS than those in the low-risk subgroup (**Figure 3C**). ROC curve showed that OS of COAD patients was perfectly predicted by the risk score [area under the curve (AUC) for 1-year survival =0.719; AUC for 3-year survival =0.751; and AUC for 5-year survival =0.697] (**Figure 3D**). A nomogram predicted the probability of 1-, 3-, and 5-year OS (**Figure 3E**). The calibration curve indicated that prediction using the nomogram was accurate (**Figure 3F**).

**Table 2:** LASSO-Cox analysis.

ID	HR	95% CI	p value	coef
PMCH	0.858	0.756-0.975	0.019	-0.152169986
LRP2	1.007	1.001-1.013	0.011	0.007619784
NAT1	0.998	0.997-0.999	0.029	-0.001180128
NKAIN4	1.005	1.002-1.008	<0.001	0.00554486
CD1B	0.978	0.958-0.999	0.043	-0.021669547

HR, hazard ratio; CI, Confidence Interval.

### Immune cell infiltration

**Figure 4:**
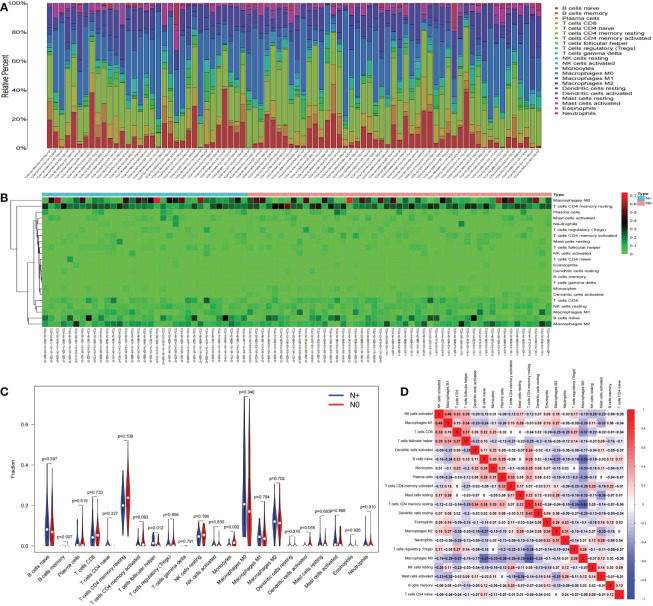
Immune cell infiltration. The composition of immune cells was assessed by the CIBERSORT algorithm in COAD **(A, B)**. The proportions of T follicular helper cells and monocytes were different in N+ and N0 group tissues **(C)**. The results of the correlation analysis of immune infiltrating cells are shown as a heatmap **(D)**. CIBERSORT, cell-type identification by estimating relative subsets of RNA transcripts; COAD, colon adenocarcinoma.

**Figure 5:**
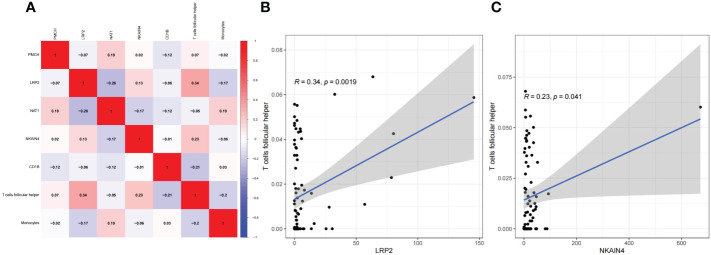
Coexpression analysis. The results of the correlation analysis of LNM-related genes expression and immune infiltrating cell are shown as a heatmap **(A)**. The correlations between LRP2 and T follicular helper cells, and NKAIN4 and T follicular helper cells **(B, C)**; LNM,lymph node metastasis.

We used CIBERSORT to calculate the proportions of 22 immune cell subsets in each COAD sample downloaded from TCGA. Barplots and heatmaps were used to visualize the data (**Figures 4A, B**). The proportions of T follicular helper cells (P=0.012) and monocytes (P=0.002) were different in the N+ group and N0 group tissues (**Figure 4C**). We evaluated the correlations in the immune cell populations (**Figure 4D**). Finally, to further explore the potential role of the PPI network and immune cell signature, we evaluated the correlations between our risk signature and immune cell populations. LRP2 and T follicular helper cells (R=0.34, P=0.0019), and NKAIN4 and T follicular helper cells (R=0.23, P=0.041) had significant correlations (**Figures 5A–C**).

### Relationship between LNM-related genes and immune checkpoints in COAD

**Figure 6:**
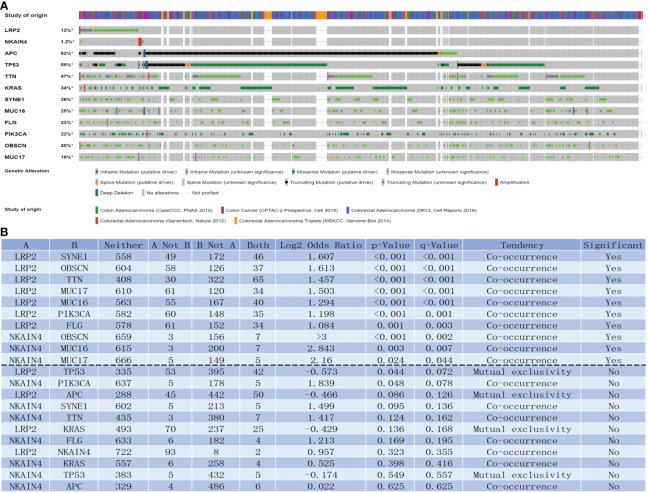
LNM-related genes with immune checkpoints in COAD. LNM-related genes and immune checkpoint alteration in COAD. Compact visualization of cases with multiple genetic alterations of LNM-related genes and immune checkpoints (origined from five studies) were individually shown by cBioPortal as indicated, including fusion, amplification, deep deletion, truncating mutation, and missense mutation **(A)**. Mutual-exclusivity analysis between LNM-related genes and multiple-immune checkpoints in COAD. The altered relationship between LNM-related genes and each immune checkpoint, such as co-occurrence and mutual exclusivity, was presented as indicated **(B)**. The detailed log2 odds ratio, P-value, Q-value, tendency, and significance were individually presented in each panel. Q-value < 0.05 was considered to be statistically significant (indicated as yes, and others as no); LNM, lymph node metastasis; COAD, colon adenocarcinoma.

Intriguingly, the genomic investigation revealed that LRP2 and NKAIN4 are involved in the alteration of immune checkpoints in COAD. The general landscape of LRP2, NKAIN4, and immune checkpoint alteration in COAD was compactly visualized, including fusion, amplification, deep deletion, truncating, and missense mutations (**Figure 6A**). The detailed relationship between LRP2, NKAIN4, and each representative immune checkpoint was individually presented, as indicated in **Figure 6B**. Of note, the LRP alteration showed a statistically significant co-occurrence rather than mutual exclusivity with extensive immune checkpoints, such as SYNE1, OBSCN, TTN, MUC17, MUC16, PIK3CA, and FLG. The NKAIN4 alteration showed a statistically significant co-occurrence rather than mutual exclusivity with extensive immune checkpoints, such as OBSCN, MUC16, and MUC17. These findings strongly indicate that LRP2 and NKAIN4 are potential coregulators of immune checkpoints in COAD.

## Discussion

Colon adenocarcinoma is one common malignancy of the digestive tract worldwide. Radiation therapy, chemotherapy and immunotherapy have been included in the treatment in addition to surgery (13, 16). Immunotherapy has become the latest research hotspot in recent years, and new immune therapeutic targets have also been identified (17, 18). Lymph node metastasis is one of the vital elements of the TNM stage. The present study identified key genes and constructed a prognostic signature to elucidate the mechanism of lymph node metastasis in COAD and shed new light on the development of immunological therapy for COAD patients.

In the present study, we identified 394 differentially expressed genes in COAD N+ group versus N0 group tissues and constructed a PPI network. Based on the components in the PPI network, the prognostic risk model for COAD patients was constructed. Ultimately, five genes(PMCH, CD1B, NAT1, NKAIN4, and LRP2, P<0.05) were incorporated into the final prognostic signatures. GO analysis revealed that DEGs were mainly enriched in immune-related pathways. Therefore, the correlation analysis between LNM-related signature and immune infiltrating cells was then investigated. We found that Tfh cells in COAD were associated with NKAIN4 and LRP2 expression.

T follicular helper (Tfh) cells are the main B cell-helper in mediating B cell differentiation in the germinal center (19–21). Tfh cells were associated with poor prognosis in Kidney Renal Clear Cell Carcinoma (22). Higher infiltrating fractions of memory Tfh cells were present in the high-risk prognostic group in hepatocellular carcinoma (23). The exhaustion of Tfh cells induced by intra-tumoral PDL1 resulted in impaired B cell function, facilitating the progression of advanced HCC (24). In addition, Tfh cells abundance improves the prognosis of patients with breast cancer or colorectal cancer (25, 26). Tfh cells are likely to be associated with the immune tolerance of tumors. We infer that the proportion of Tfh cells failed to serve as independent prognostic markers because COAD tissue with lymph node metastasis may be highly immune-tolerant, thereby inhibiting the immune effect of B cells. This mechanism contributes to tumor invasion and metastasis.

PMCH(promelanin-concentrating hormone) may act as a neurotransmitter or neuromodulator in neuronal functions that regulate goal-directed behavior, such as food intake and general arousal (27). In renal clear cell carcinoma, the low expression of PMCH is significantly related to higher tumor stages, more chances of metastasis, and poor prognosis (28). This study found that the high expression of the PMCH gene is a good prognostic factor for the survival of COAD patients.

Findings have shown that leukemia and several solid tumors contain a rare subpopulation of cells with specific surface CD markers that has the potential to self-renew, sustain cancer growth, and predict prognosis (29, 30). Low CD1B expression correlated with poorer biochemical recurrence-free survival in prostate cancer. CD1B confers prostate cancer progression and may help improve clinical prognostic stratification (31). The TME-related signature consisting of CD1B(HR 0.027, 95%CI 0.002-0.284, p=0.003) is effective to predict survival and to provide individualized treatment for COAD patients (32). The high expression of CD1B exhibited a significant correlation with longer OS in colorectal cancer (33). Guo et al. found that CD1B, which is involved in resting and activated dendritic cells, may be potential novel biomarkers for immunotherapy (34). The current research results on CD1B are consistent with the results of our research, indicating that the high expression of CD1B is a good prognostic factor for COAD. Moreover, CD1B is related to resting and activated dendritic cells and may be used as an immunotherapy target.

The NAT1 gene, located on chromosome 8p22, encodes one of two human enzymes known to metabolize arylamine- and hydrazine-type drugs (35). In several cancers, the expression of NAT1 is associated with cell proliferation *in vitro* and with survival *in vivo* (36). A previous study has reported that low NAT1 expression resulted in a distinctly poor response to chemotherapy in breast cancer patients (37). NAT1 showed significant positive correlations with OS and could be considered to be protective genes in COAD (38). In COAD, NAT1 has been found to be enriched in the caffeine metabolism pathway (39). NAT1 may function as a tumor suppressor gene, and that a high level of methylation could reduce the mRNA expression of this gene in COAD tissues (40).

The high expression of NKAIN4 and LRP2 is related to the poor prognosis of COAD patients and has a co-expression relationship with the immune infiltrating cells related to lymph node metastasis.

NKAIN4 is named for its interaction with the β1 subunit of Na(+)/K(+) transporting ATPase (sodium-potassium pump). In recent years, the role of the sodium-potassium pump in tumors has attracted the attention of related scholars (41). As a transmembrane protein complex, a sodium-potassium pump can remove 3 Na+ from the cell by decomposing 1 ATP and moving 2 K+ into the cell. This function is necessary for cells to maintain osmotic pressure and establish an electrochemical gradient (42). In recent years, studies have found that sodium-potassium pumps also act as signal transduction molecules, playing an important role in cell growth, differentiation, survival, metastasis, invasion, and intercellular and cell-to-interstitial connections forming a scaffold for protein interactions (43). The role of the sodium-potassium pump in tumors has attracted widespread attention, and many studies have found that its activity is enhanced in a variety of tumors (44). Some scholars have tried to use the sodium-potassium pump subunit as a tumor biomarker. It is proposed that the subunit is highly expressed in lung cancer, kidney cancer, colorectal cancer, and medulloblastoma, and the expression of β subunit is increased in cervical cancer and colorectal cancer (45–47). We speculate that in COAD, NKAIN4 further promotes the activity and function of the sodium-potassium pump by activating the expression of the β1 subunit, thereby promoting tumor proliferation and progression. In our research, we also found that the expression of NKAIN is correlated with Tfh cells, although the correlation between them is not so strong(R=0.23, P<0.05), suggesting that there may be some regulation between NKAIN and Tfh cells in COAD.

LRP2(LDL receptor-related protein 2), a member of the LDL receptor family, is a crucial regulator in the sonic hedgehog pathway, an important developmental process (48). Megalin, encoded by the gene LRP2, is an abundantly present cell surface protein in the kidney that binds DBP to mediate the internalization of 25(OH)D into the cytosol (49). Calcitriol may prevent cancer progression by multiple levels, including reducing cell proliferation, increasing cell differentiation, and apoptosis (50–52). Calcitriol suppresses glycolysis and cell growth in human colorectal cancer cells, suggesting an inhibitory role of the biologically active form of vitamin D in colorectal cancer progression (53). Therefore, the reduction of vitamin D may promote lymph node metastasis in COAD. The SNP of LRP2 can cause a decrease in vitamin D (54), but we did not find a significant difference in mutation frequency in our sample (**Figure S1**). Thus, we infer that the relevant mechanism between LRP2 and COAD lymph node metastasis may not be related to reducing vitamin D but to other effects of LRP2, such as the reuptake of many ligands and signal transduction. Among other tumors, there are also some studies on LRP2. Higher expression of LRP2 was reported as a favorable prognostic factor in renal cell carcinoma (55). Germ-line polymorphisms in the LRP2 gene may be associated with an increased risk for recurrence in prostate cancer (56). Somatic mutations in LRP2 have been identified in gastric cancer (57). It can be seen that the research on the invasion and prognosis of LRP in tumor tissues is still inconclusive. This study found that LRP2 is related to the worse survival rate of COAD patients and is related to Tfh cells.

In COAD tissues with lymph node metastasis, there are more infiltrating Tfh cells. Moreover, the LRP2 gene and NKAIN gene have a significant correlation with Tfh cells. We preliminarily speculate that COAD tissues with lymph node metastasis may have a higher level of immune tolerance, which may cause increased Tfh cells infiltration. Further studies are warranted in connections between genes and Tfh cells. We also found that the NKAIN4 and LRP2 alteration showed a statistically significant co-occurrence rather than mutual exclusivity with extensive immune checkpoints. These findings strongly indicate that LRP2 and NKAIN4 are potential coregulators of immune checkpoints in COAD.

Our study shows that LRP2 and NKAIN4 genes can regulate the lymph node metastasis of COAD, and may regulate the infiltration of Tfh cells to mediate immune tolerance. LRP2, NKAIN4 and Tfh cells can show the migration ability of COAD cells, so the prognosis of patients with COAD can be predicted clinically by detecting the above three molecules. In addition, targeted drugs against LRP2 and NKAIN4 may also improve the immune tolerance of cancer cells, thereby reducing the proliferation, invasion and migration capabilities of cancer cells, and improving the prognosis of patients.

This study mainly has the following shortcomings: 1. The data used for analysis comes from public databases, there is no uniform criterion for data standardization, and the lack of clinical data in some patients may cause bias. 2. No further experimental verification. 3. There are few studies on LRP2, NKAIN4, and Tfh cells in COAD, and the mechanism of action cannot be fully elucidated. The present study has the following strengths: 1.To the best of our knowledge, this is the first study on the relationship between LNM-related genes and immune infiltrating cells in COAD, which is innovative. 2. The clinical prediction model constructed has good predictive performance. 3. Our findings also provide ideas for future targeted therapy and immunotherapy.

This study constructed a prognostic model of genes related to lymph node metastasis in COAD and found that PMCH, CD1B, NAT1, NKAIN4, and LRP2 have prognostic, predictive values. Then, we performed a correlation analysis between genes with prognostic significance and differential immune infiltrating cells in COAD tissues. It is found that LRP2 and NKAIN4 are related to the infiltration of Tfh cells in COAD. Further studies have found that LRP2 and NKAIN4 are potential coregulators of immune checkpoints. This study helps to clarify the mechanism of lymph node metastasis in COAD, and in-depth research is needed to demonstrate and clarify the detailed mechanism.

## Data availability statement

The original contributions presented in the study are included in the article/**Supplementary Material**. Further inquiries can be directed to the corresponding author.

## Author contributions

HXZ, GBZ: Conception, Design of the work, Analysis and Writing. GWZ: Supervision, Reviewing and Editing. JXY: Writing, Reviewing and Editing. All authors contributed to the article and approved the submitted version.

## Supplementary material

Supplementary Figure 1 The mutation of LRP2 mRNA in different groups of samples, in which the horizontal axis represents the samples of different groups, the vertical axis represents the distribution of the mRNA mutation, where different colors represent different mutation types, and the right side represents significance, P value is the test method.
