# Supplementary figures and images for: Identification of lymph node metastasis-related genes and patterns of immune infiltration in colon adenocarcinoma
Source: Front Oncol. 2023 Jan 16;12:907464. doi: 10.3389/fonc.2022.907464 (PMC9884978; doi:10.3389/fonc.2022.907464)

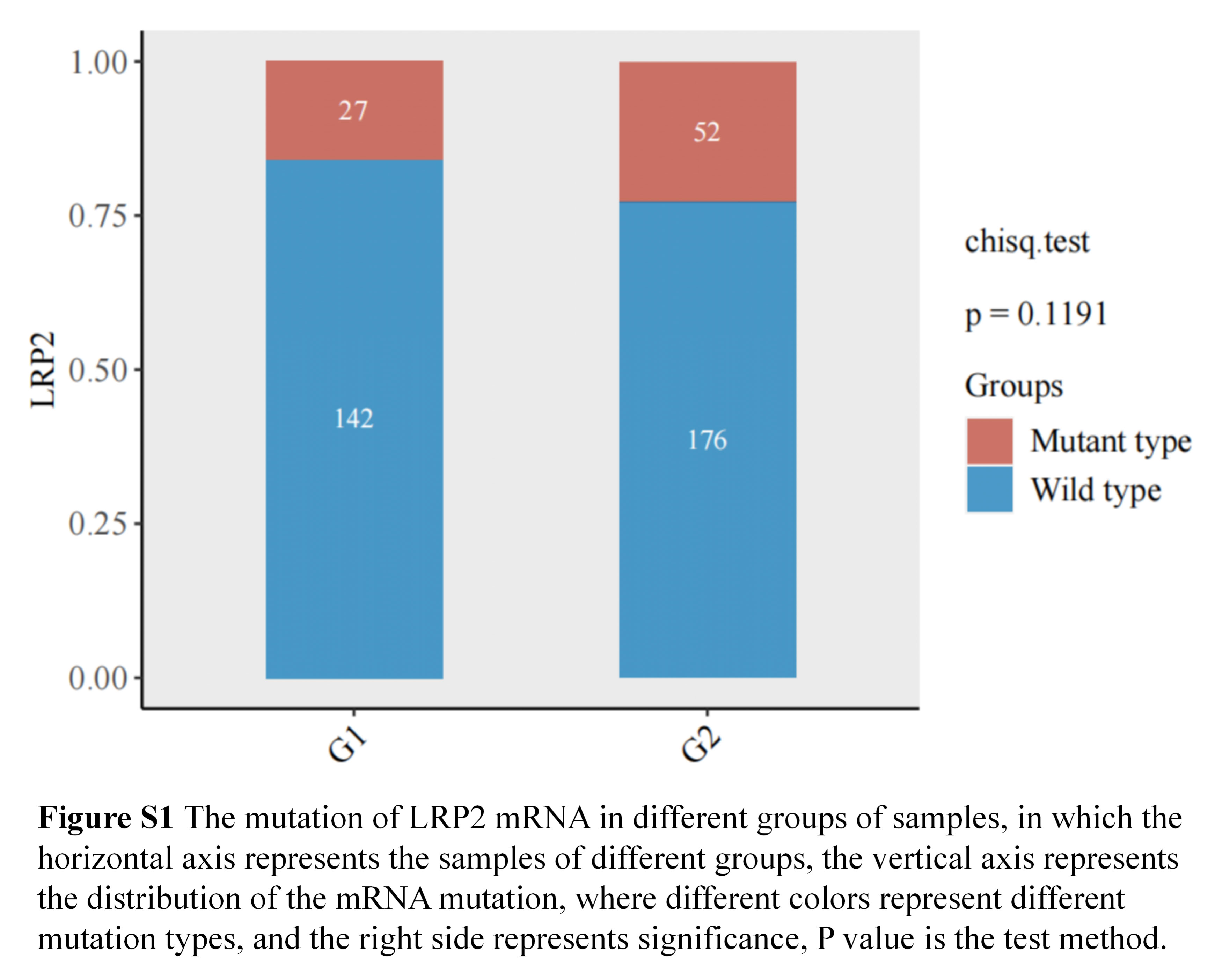

Supplement: Supplementary Figure 1 — The mutation of LRP2 mRNA in different groups of samples, in which the horizontal axis represents the samples of different groups, the vertical axis represents the distribution of the mRNA mutation, where different colors represent different mutation types, and the right side represents significance, P value is the test method. [file Image_1.jpeg]
